# Common side effects of MDMA-assisted psychotherapy

**DOI:** 10.1038/s41386-024-01873-8

**Published:** 2024-05-13

**Authors:** Conor H. Murray

**Affiliations:** Department of Psychiatry and Biobehavioral Sciences, University of Los Angeles, California, Los Angeles, CA USA

**Keywords:** Signs and symptoms, Psychology

MDMA-assisted psychotherapy has emerged as a potential treatment for psychiatric illnesses, with findings from clinical trials in the last decade demonstrating safety and efficacy in the treatment of posttraumatic stress disorder (PTSD), among other psychiatric indications. Earlier this year (2024), the Food and Drug Administration in the United States granted priority review for a new drug application involving MDMA-assisted psychotherapy for PTSD, which followed their Breakthrough Therapy designation for MDMA-assisted psychotherapy in 2017 [1]. In addition, the Australian Therapeutic Goods Administration has authorized MDMA prescriptions for PTSD, making Australia the first country to recognize medical uses for MDMA [2]. Beyond PTSD, evidence from clinical trials indicates that MDMA-assisted psychotherapy is safe, tolerable, and effective in patients with alcohol use disorder, autism, and end-of-life anxiety [3].

The safety profile of MDMA, without psychotherapy, is well characterized in healthy research participants in Phase 1 studies. In an analysis across nine Phase 1 studies from 2017, single-dose administration of MDMA near to 100 mg was deemed safe for healthy participants relative to placebo [4]. Those findings opened a path for future studies of MDMA as an adjunct to psychotherapy in controlled clinical settings. Now, clinicians and scientists recognize that the safety profile of MDMA with psychotherapy included, as found in Phase 2 and 3 studies with patient populations, is still largely uncharacterized. The absence of available evidence has contributed to concern by some psychiatrists, such as Steve Kisely in Australia, who has questioned whether his country’s recent policy moves are premature [2].

Here, I provide research highlights from the recent report by Colcott et al. [3], who conducted a timely meta-analysis and systematic review of the side effects of MDMA-assisted psychotherapy in Phase 2 and 3 studies across psychiatric indications. Side effects were defined inclusively across the range of reported safety outcomes, whether reported as adverse events or spontaneously as off-target reactions to the drug. Eight randomized placebo-controlled trials were included in their meta-analysis, while five additional studies, which did not have a control group, were also included in their systematic review. The authors extracted data related to the study population, MDMA dose(s), side effects, and the timing of side effect assessment. Notably, data from therapeutic outcomes in these studies were not extracted. This is somewhat unfortunate, as therapeutic outcome data are necessary to gain insight into the relationship between side effects and therapeutic outcomes, including whether doses that do not induce side effects are nonetheless sufficient to produce meaningful therapeutic outcomes. The review focuses instead on the side effect data regardless of therapeutic outcomes, examining both the quality of the side effect data extracted and the risk of bias in reporting.

Common side effects that Colcott et al. found during MDMA-assisted psychotherapy sessions, which were not reported as adverse events, included jaw clenching and anxiety. Jaw clenching was more likely to occur at 125 and 150 mg doses. Other side effects did not reach significance in the meta-analysis. Overall, 30% of controls reported side effects relative to 45% of participants receiving MDMA-assisted psychotherapy, or 1.7 times greater odds of experiencing a side effect relative to control. Similarly, within 7 days following the MDMA, 31% of controls reported side effects relative to 46% of MDMA participants, 1.6 times greater odds. Within 7 days, no specific side effects reached significance in the meta-analysis.

The odds of side effects reported as adverse events were higher in Phase 3 studies after MDMA but not in Phase 2 studies. In the Phase 3 studies, 5% of participants treated with placebo had an adverse event, relative to 16% after MDMA-assisted psychotherapy, or 3.5 times greater odds. MDMA specifically increased the odds of adverse events with jaw clenching and muscle tightness, decreased appetite, nausea, excessive perspiration, feeling cold, restlessness, blurred vision, dilated pupils, uncontrolled eye movements, feeling jittery, non-cardiac chest pain/discomfort, and chills. There was no difference in the odds of withdrawal from participating in Phase 2 or 3 studies after MDMA.

In the assessment of the quality of the data and risk of bias in the reporting, seven of the eight studies in the meta-analysis were rated as “high risk of bias,” with one rated as “some concerns.” The certainty of the evidence was also found to be low, with all side effect outcomes in Phase 2 studies found to have “very low” certainty of evidence, and two of the three side effect outcomes in Phase 3 studies found to be “very low,” with one rated as “moderate.”

Despite the overall low quality of evidence and high risk of bias, the authors provided the first comprehensive meta-analysis and systematic review of MDMA-assisted psychotherapy across psychiatric indications. Side effects were relatively modest relative to placebo controls. The side effects were also transient, occurring primarily under the supervision of medical professionals in controlled clinical settings. Given the overall quality of the evidence however, the authors advise caution in interpreting the findings. The relatively small number of studies with varying doses also limited granular insights into the role of dose in the reported effects. Across the eight placebo-controlled trials, doses ranged from 50 to 150 mg, with side effects appearing to emerge at 75 mg. For the field, an important next step will be to determine how side effects are related to therapeutic outcomes across doses. Whether certain side effects during MDMA-assisted psychotherapy may predict the presence or absence of therapeutic responses will inform best practices that maximize therapeutic benefits while minimizing unnecessary risks.
